# Generalized Epileptic Seizures in Fibrodysplasia Ossificans Progressiva Harboring a Recurrent Heterozygous Variant of the *ACVR1* Gene (R206H)

**DOI:** 10.1155/crig/9569275

**Published:** 2024-12-17

**Authors:** Kenichi Mishima, Hiroshi Kitoh, Anna Shiraki, Kenta Sawamura, Yasunari Kamiya, Masaki Matsushita, Shiro Imagama

**Affiliations:** ^1^Department of Orthopaedic Surgery, Nagoya University Graduate School of Medicine, 65 Tsurumai-cho, Showa-ku, Nagoya 466-8550, Aichi, Japan; ^2^Department of Orthopaedic Surgery, Aichi Children's Health and Medical Center, 7-426 Morioka-cho, Obu 474-8710, Aichi, Japan; ^3^Department of Pediatrics, Nagoya University Graduate School of Medicine, 65 Tsurumai-cho, Showa-ku, Nagoya 466-8550, Aichi, Japan

**Keywords:** absence seizures, *ACVR1*, fibrodysplasia ossificans progressiva, heterotopic ossification, idiopathic generalized epilepsy, juvenile myoclonus epilepsy, myoclonus

## Abstract

**Background:** Fibrodysplasia ossificans progressiva (FOP) is an ultra-rare disorder caused by heterozygous *ACVR1* pathogenic variants and is characterized by both progressive heterotopic ossification of the soft tissues and congenital malformations of the great toe. In addition to pathological skeletal metamorphosis, patients with FOP experience diverse neurological symptoms such as chronic pain and involuntary movements; however, little is known about the association between FOP and epileptic seizures.

**Methods:** We report the case of a young boy with FOP who sustained multiple major fractures due to epileptic loss of consciousness.

**Results:** Based on generalized electroencephalographic abnormalities and the presence of myoclonic movements, the patient was diagnosed with juvenile myoclonic epilepsy. The absence of seizures was well-controlled with valproic acid, whereas occasional abrupt myoclonic movements of the hands and feet persisted.

**Conclusion:** This case expands our understanding of the phenotypic diversity of FOP and the functional versatility of *ACVR1*-mediated bone morphogenetic protein (BMP) signaling.

## 1. Introduction

Fibrodysplasia ossificans progressiva (FOP) is a debilitating genetic condition characterized by congenital malformations of the great toe and progressive heterotopic ossification (HO) of soft tissues including skeletal muscles, ligaments, tendons, fasciae, and aponeuroses [1]. FOP is caused by heterozygous gain-of-function pathogenic variants in the cytoplasmic domain of activin receptor-like kinase 2 (ALK2)/activin A receptor type 1 (*ACVR1*), one of the four ligand-activated bone morphogenetic protein (BMP) type I receptors [2]. Mechanistically, mutated *ACVR1* confers ligand-dependent overactivation and ligand-independent constitutive activation on canonical BMP-Smad signaling [3]. This conventionally accepted pathomechanism of FOP has been considered to conflict with actual clinical pictures, such as postnatal episodic HO and normal development and growth. Recent investigations have revealed a novel mechanism for HO: the mutated *ACVR1* receptor gains the ability to misrecognize activin A, a competitive antagonist of the wild-type *ACVR1* receptor and a molecule that normally transmits TGF-*β* signaling [4]. This unexpected ligand activin A transduces aberrant BMP signaling coupled with physiological TGF-*β* signaling, leading to ectopic bone formation typically preceded by inflammatory painful subcutaneous swelling called “flare-up” [5]. Several reagents that can neutralize activin A or inhibit its downstream signaling are currently under development to prevent HO [6]. The mammalian target of rapamycin (mTOR) signaling pathway has been identified as an activin A-induced critical pathway for the aberrant chondrogenesis of mesenchymal stem cells [7], and a clinical trial of the mTOR inhibitor sirolimus (rapamycin), aimed at the preservation of physical function and suppression of HO, was implemented in Japan (https://upload.umin.ac.jp/cgi-open-bin/ctr/ctr_view.cgi?recptno=R000032495).

In addition to these pronounced musculoskeletal abnormalities, neurological dysfunction is another hallmark of FOP that may continuously afflict patients with sensory symptoms, such as recurrent severe headaches, neuropathic pain, and other sensory abnormalities [8]. Less commonly, some patients present with nonsensory symptoms, including recurrent seizures, movement disorders such as tremors and myoclonus, and traumatic brain or spinal cord injuries [9]. To our knowledge, there have been no previously identified cases of absence seizures in FOP patients.

In this report, we describe the case of a patient with FOP who sustained multiple long-bone fractures due to idiopathic generalized epilepsy with falling. The patient's parents consented to the submission of the data concerning this case for publication.

## 2. Clinical Report

A Japanese boy was delivered at 38 weeks of gestation by cesarean section because of breech presentation. His father was diagnosed with FOP based on pathognomonic skeletal features and a pathogenic variant of the *ACVR1* gene (c. 617G > A; p. R206H). At the age of 10 months, the boy was referred to our institution with a bilateral congenital hallux valgus deformity. The patient was confirmed to have FOP owing to the presence of the same recurrent variant in the *ACVR1* gene as his father. This was the first reported case of familial FOP in Japan [10].

**Figure 1:**
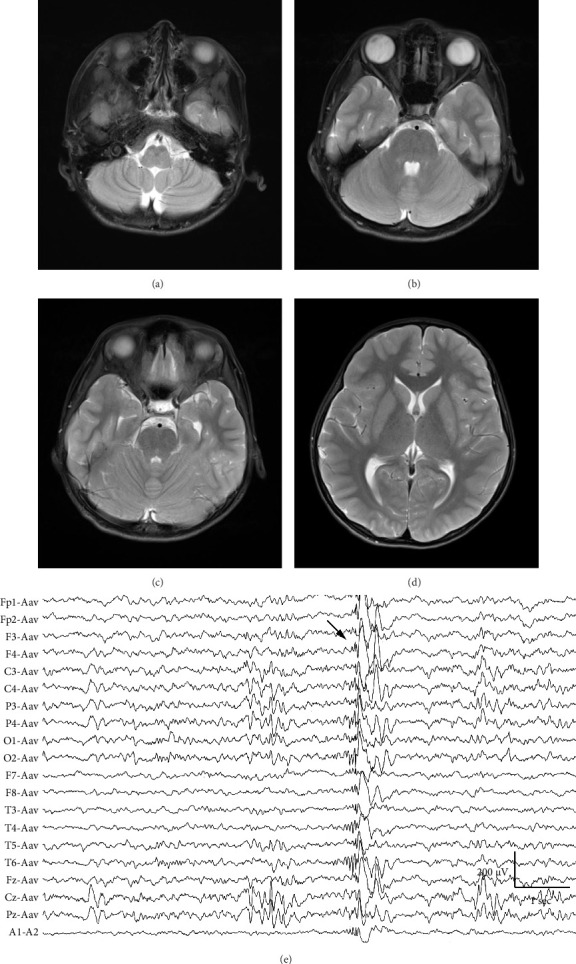
Diagnostic work-up of syncope with brain MRI (a–d) and electroencephalography (e). Axial T2-weighted images at the level of the medulla (a), cerebellum (b), pons (c), and basal ganglia (d) showing no white or gray matter abnormalities. (e) Example of interictal discharges on the average reference montage of a drowsy EEG. Right frontal dominant irregular, diffuse spike-waves, and polyspike-waves are shown (arrow).

**Figure 2:**
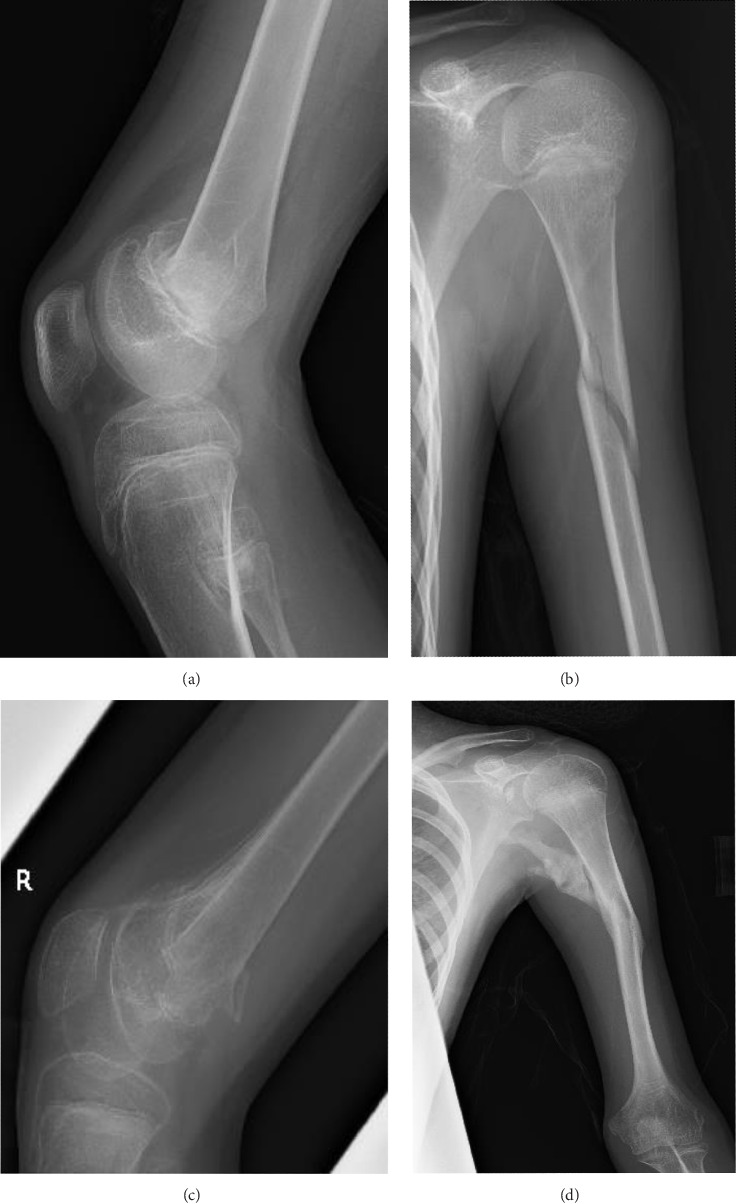
Bone fractures in association with generalized seizures (a) A radiograph of the right knee made at the time of injury showing a displaced Salter–Harris type II physeal fracture of the distal femur. (b) A radiograph of the left humerus made at the time of injury showing a mid-shaft spiral fracture of the humerus. (c) A radiograph of the right knee made 6 months after the injury showing sagittal rotational malunion of the distal femur without apparent HO. (d) A radiograph of the left humerus made 6 months after the injury showing the proper alignment and well union of the fracture. Note abundant heterotopic bones connecting the mid-shaft humerus and the glenoid neck of the scapula.

At 12 years of age, he was found sitting with clouded consciousness in the bathtub. His extremities and trunk appeared weak with no convulsive movement or clenched teeth. His parents immediately called for emergency services because he moaned and failed to respond to their calls. His consciousness was fully recovered upon arrival at the emergency department. No abnormal vital signs or neurological findings were observed except tachycardia (145 beats/min) and tachypnea (26 breaths/min). There was localized mild swelling of the right knee and intolerable pain even with slight flexion. The patient was then hospitalized for further evaluation. At that time, the patient had participated in the rapamycin trial. Brain magnetic resonance imaging (MRI) revealed no acute cerebral infarction, supratentorial or subtentorial hemorrhage, or gray or white matter lesions (Figure 1(a)). No occlusive or narrowed lesions were detected in the carotid and vertebral arteries or their intracranial branches on MR angiography. Holter electrocardiographic monitoring revealed no abnormalities associated with the cardiac syncope. Radiographs revealed a displaced Salter–Harris type II physeal fracture [11] of the distal femur (Figure 2(a)). Three days after admission, electroencephalography (EEG) was performed, which was interpreted as the absence of spikes, sharp waves, and slow waves. The patient was discharged with his right knee immobilized in a long-leg cast. Three weeks after the fainting episode, a second EEG was conducted, revealing sporadically occurring right frontal dominant irregular, diffuse spike-waves and polyspike-waves during both awake and sleep states (Figure 1(b)). The patient was diagnosed with idiopathic generalized epilepsy and treated with valproic acid. One and a half months after the epileptic seizure, the patient sustained a mid-shaft spiral fracture of the left humerus due to a fall faint episode (Figure 2(b)), which was treated conservatively with sling immobilization.

At present, 3 years have passed since the initiation of valproic acid treatment. The patient still occasionally showed involuntary myoclonic movements in the distal upper or lower limbs. The epiphyseal fracture of the right knee healed without any new HO (Figure 2(c)), whereas the abundant heterotopic bones that formed between the left humerus and scapula made the shoulder joint virtually immobile (Figure 2(d)).

## 3. Discussion

According to a global survey of FOP patients regarding neurological symptoms, four out of 168 patients (2.4%) reported their experiences of recurrent seizures, whereas the type of those seizures or whether there is the coexistence of absence seizures is unknown because of the patient self-reporting questionnaire-based survey [8]. Here, we describe the case of a patient with FOP who developed idiopathic juvenile epilepsy. To the best of our knowledge, this is the first report of FOP complicated by EEG-confirmed idiopathic generalized epilepsy. Although the association between FOP and seizures has hardly been discussed, the possibility of absence seizures merits consideration in traumatized patients with FOP. Contrary to the common belief, only a small percentage (10%) of fractures produced adjacent HO and appreciable loss of motion in FOP [12], whereas one of the affected joints was completely immobile in this case because of progressive HO.

A wide range of unusual neuropsychiatric symptoms develop in FOP patients, such as persistent pain, mild mental retardation, and involuntary movements; however, their etiologies remain elusive [8, 9]. Recently, structural and white matter abnormalities in the central nervous system (CNS) have been implicated in the pathogenesis of neurological symptoms [13–15]. Histological and immunohistochemical studies using genetically engineered *Acvr*1^*R*206*H*/+^ knock-in mice detected multiple areas of demyelination in the brain and spinal cord, which appear as T2-weighted hyperintense lesions on MRI. MRI of the brain and spinal cord in adult and pediatric patients with FOP showed T2-weighted hyperintense lesions scattered in the periventricular white matter, subcortical white matter, pons, cerebellum, and spinal cord. Since upregulated canonical BMP-Smad signaling inhibits the differentiation of oligodendrocyte precursor cells (OPCs) into myelin-forming oligodendrocytes (OLs) while enhancing the generation of astrocytes [16], it is plausible that impaired remyelination or dysmyelination occurs in FOP, where constitutive activation of BMP signaling occurs. If this is true, it is unclear what external stimuli induce defects in OL differentiation and myelination in some parts of the CNS, as shown in the previous studies [13–15]. One candidate molecule is the coagulation factor fibrinogen, which activates *ACVR1*-induced Smad-dependent BMP signaling in OPCs [17]. Less frequently, patients with inflammatory demyelinating disorders of the CNS, such as multiple sclerosis can experience acute symptomatic seizures and chronic epilepsy [18], however, in this case, no CNS involvement was detected on MRI regarding white matter lesions or brainstem dysmorphisms. It remains uncertain whether CNS demyelination is relevant to generalized epilepsies due to the rarity of FOP and the limited resolution and sensitivity of current MRI imaging.

In summary, we present an unreported case of generalized epileptic seizures in a young boy with FOP who sustained multiple major fractures due to epileptic loss of consciousness. This case expands the phenotypic spectrum of FOP with respect to neuropsychiatric conditions. Clinical considerations of the unusual association between FOP and generalized epilepsy are needed when treating traumatized individuals with FOP.

## Data Availability

The data that support the findings of this study are available from the corresponding author upon reasonable request.
